# Estimation of the Incidence of Bacterial Vaginosis and other Vaginal Infections and its Consequences on Maternal/Fetal Outcome in Pregnant Women Attending an Antenatal Clinic in a Tertiary Care Hospital in North India

**DOI:** 10.4103/0970-0218.66855

**Published:** 2010-04

**Authors:** Indu Lata, Yashodhara Pradeep, Sujata, Amita Jain

**Affiliations:** Department of Maternal and Reproductive Health, Sanjay Gandhi Post Graduate Institute of Medical Sciences, Lucknow, UP, India; 1 Department of Obstetric & Gynaecology, K. G. Medical University, Lucknow, UP, India; 2 Department of Microbiology, K. G. Medical University, Lucknow, UP, India

**Keywords:** Bacterial vaginosis, other vaginal infection, pregnancy outcome, UTI

## Abstract

**Aims:**

This study was undertaken to estimate the incidence of bacterial vaginosis (BV) and other vaginal infections during pregnancy and its association with urinary tract infections (UTI) and its consequences on pregnancy outcome, maternal and fetal morbidity and mortality.

**Settings and Design:**

Prospective cohort study.

**Materials and Methods:**

The present prospective cohort study was conducted on 200 women attending the antenatal clinic (ANC) of a tertiary hospital. All pertinent obstetric and neonatal data covering antenatal events during the course of pregnancy, delivery, puerperium and condition of each newborn at the time of birth were collected. BV was detected by both Gram stain and gold standard clinical criteria (Amsel’s composite criteria).

**Statistical analysis used:**

Data were analyzed using SPSS version 9. Fischer’s exact test, chi square tests and Student’s’ test has been used for analysis. The probability of 5% was considered as significant for continuous variables such as age, period of gestation and birth weight. Odds ratio (OR) and confidence interval (CI) with 95% probability were determined.

**Results:**

The incidence of bacterial vaginosis was 41 in 200 patients. Adverse outcomes such as preterm labor, PROM and fetal complications were found more in pregnant women who had bacterial vaginosis (*N*=41), bacterial vaginosis with UTI (*N*=14) as compared to those without bacterial vaginosis (*N*=118).

**Conclusions:**

The incidence of poor pregnancy outcome was higher in bacterial vaginosis with UTI. Prevention of BV and UTI is cost effective to minimize the pregnancy-related complications and preterm labor to decrease in perinatal and maternal mortality and morbidity. We recommend all antenatal patients should be screened for the presence of bacterial vaginosis, other infections and UTI.

## Introduction

Bacterial vaginosis (BV) is a condition in which the normal, lactobacillus-predominant vaginal flora is replaced with anaerobic bacteria, gardnerella vaginalis, and mycoplasma hominis.(1) Bacterial vaginosis has been associated with premature rupture of membranes,(2,4) preterm delivery,(2–7) infection of the chorion and amnion,(8) histologic chorioamnionitis,(8) infection of amniotic fluid, intrauterine death. (9–11) In other reports, the microflora associated with bacterial vaginosis, including anaerobic Gram-negative rods, G. vaginalis, and M. hominis, has been linked to preterm delivery.(12–14)

This suggests that it may be possible to prevent a proposition of preterm births by screening women for bacterial vaginosis and eradicating it early in pregnancy. BV is detected by Gram stain (Spiegel criteria,(15) Nugent criteria(16)) and accepted Gold standard criteria (Amsel’s composite criteria).(17) In 2000, for the first time, there was a report that women suffering from (BV) are at greatest risk of UTI than others with increased risk of HIV and STD.(11,18)

## Materials and Methods

The present double blind, prospective study was conducted on 200 pregnant women attending the antenatal clinic, after taking approval from institutional ethical committee and informed written consent from the patients. All pertinent obstetric and neonatal data covering the course of pregnancy, delivery and the puerperium, as well as condition of each newborn were collected, under the following headings: abortion, premature rupture of membrane(PROM) and preterm premature rupture of membrane (PPROM), spontaneous/induced labor, period of gestation at the time of delivery, birth weight of the baby, maturity, high risk factor for mother, obstetrical complications and any other relevant information. All questionnaires were administered and all examinations and microbiologic procedures were performed according to a standardized protocol. Women were enrolled in the study during routine prenatal visits of gestation 10 weeks to term; for each woman, a medical, obstetrical, sexual, and social history was taken and cultures of the vagina was obtained.

### Collection of specimens

After assurance of patient, clean unlubricated speculum was passed into the vagina to see the condition of the vaginal wall, cervix and nature of the discharge. ‘Whiff’ test was done for ‘Fishy odor’ with collected discharge on speculum. First swab sample was taken from the posterior vaginal fornix aseptically tested for pH and then made slide for Gram staining. The second swab was put into a sterile test tube for culture. The urine sample was collected by midstream and clean catches method in a sterile container for analysis and culture.(18) The methods used to detect microbiologic organisms have been described elsewhere.(19)

### Evaluation of vaginal smears

BV was detected by both Gram stain (Spiegel criteria, Nugent score)(20) and accepted Gold standard criteria (Amsel’s composite criteria), defines bacterial vaginosis as being present if three of the following criterion are found.(19) (1) homogenous vaginal, discharge, (2) vaginal pH greater than 4.5, (3) positive ‘Whiff’ test and (4) the presence of clue cells on wet microscopy of vaginal fluid.(17)

### Diagnosis of bacterial vaginosis

Patients, who fulfilled 3 out of 4 clinical criteria (Amsel *et al*), were diagnosed as bacterial vaginosis (BV). On evaluation of Gram stain, women could be diagnosed as BV (S) or Non BV (Non-Bacterial Vaginosis), and based on criteria suggested by Spiegel *et al*., women could be diagnosed as BV (N) intermediate BV (N) or Non BV on the basis of criteria put forward by Nugent *et al*.

**Graph 1:**
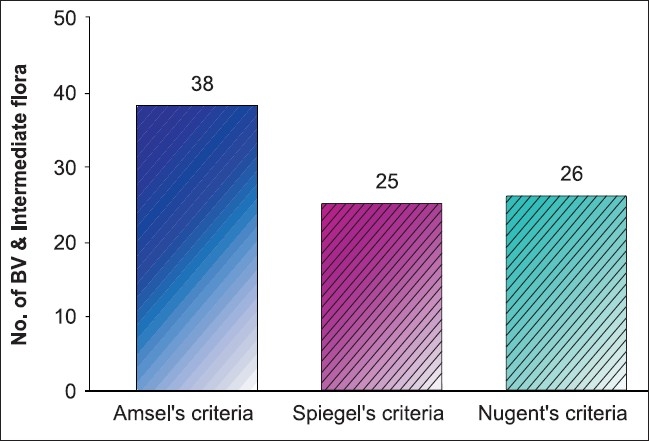
Dignosis of bacterial vaginosis by clinical criteria (Amselæs criteria) and gram stain criteria (Spiegelæs criteria, nugentæs criteria)

**Graph 2:**
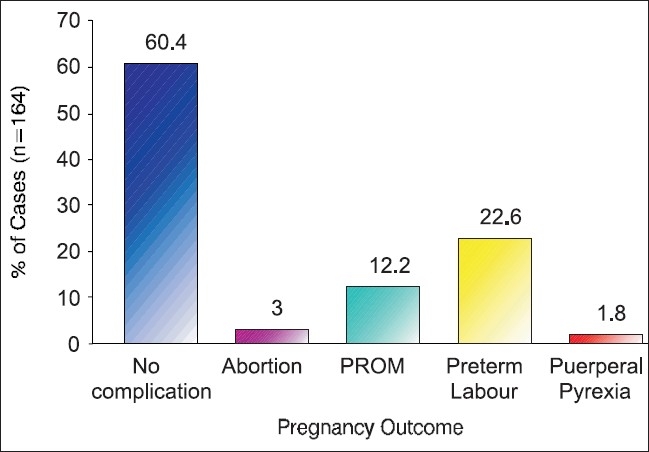
Showing pregnancy outcome of studied patients (N=164)

Observations and results are shown in [Table 1–4] and [Graphs 1,2].

**Table 1:** Incidences of different vaginal infection

Vaginal Infections	Frequency (*N*=200)
Bacterial vaginosis	38 *
Bacterial vaginosis + Trichomonas vaginalis	0
Bacterial vaginosis + Candidiasis	3
Candidiasis	9
Trichomonas vaginalis	0
Other infections	0

* *P*=0.000 i.e. <0.001 (highly significant),

*X*^2^=45.86; d.f. 2;

**Table 2:** Distribution of age, parity, socio-economic status, and gestational age

**(A) Age-wise distribution of B.V. associated with UTI**	**Frequency (*N*=14>**
18-27 *years	11*
28-35 years	3
>35 years	0

**(B) Parity-wise distribution**	**Frequency (*N*=14)**

P0+0*	8*
P1+0	3
P2+0	1
P3+0	0
P0+1	1
P0+2	0
P1+1	1

**(C) Socio-economic status of BV positive**	**Frequency (*N*=41)**

Upper	1
Upper-middle	1
Lower-middle	1
Upper-lower	10
Lower*	28*

**(D) Gestational age at which sample taken**	***N*=200 frequency**	**B.V Positive**

0-10 weeks	0	0
11-20	62	14
21-30	90	19
31-40	43	8

* ‘*P*’ value was found to be statistically significant

**Table 3:** Adverse pregnancy outcome with BV, without BV and with BV associated with UTI

	Without BV (*n*=118)	BV only (*n*=41)	Intemediate (*n*=5)	BV with UTI (*n*=14)
Abortion	2	3 *	0	1
PROM	9	11 *	0	3
Preterm labor	15	22 *	0	9
Conservatively	8	4		3
Delivered	6	19		6
Puerperal pyrexia	1	2*	0	1
Birth weight				
2.5 kg	100	16	5	10
2.0-2.5 kg	18	24	0	4
<2.0 kg	0	1	0	0

* ‘*P*’ value was found to be statistically significant compared to without B.Vaginosis

**Table 4:** Incidence of bacterial vaginosis, UTI and their association

*N*=200	No	%
Bacterial vaginosis present	41*	20.5
Bacterial vaginosis absent	159	79.5
Urinary tract infection present	51*	25.5
Urinary tract infection absent	149	74.5
Bacterial vaginosis associated with UTI in 41 patient with BV	14	34.1
Bacterial vaginosis without UTI	27	65.85

* ‘*P*’ value was found to be statistically significant

## Discussion

Out of 200 patients enrolled, 164 patients could be followed during ANC to delivery at our institute, and the remaining 36 patients who were having no vaginal infection on investigation either not turned back or delivered elsewhere. The incidence of types of infection in 200 patients is comparable with Govender *et al*(21) and Levett *et al*(22) [Table 1].

The incidence of bacterial vaginosis was most common in the age group of 18–27 years and in primipara between the gestational ages 11–20 weeks comparable with Cristiano.(23) The incidence of bacterial vaginosis was most common in lower socio-economic status (*P*=0.0477) [Table 2].

The condition of cervix on per speculum examination was evaluated. The incidence of bacterial vaginosis with unhealthy cervix was in 8 patients (66.7%) in comparison to bacterial vaginosis with healthy cervix in 33 patients (17.6%), statistically significant (*P*=0.016) [Table 2].

In this study, the criteria (Amsel’s, Spiegel, Nugent *et al*) followed for the diagnosis of bacterial vaginosis in which Amsel’s clinical criteria [38/46 (82.6%)] was statistically highly significant compared to two other ones [Graph 1]. The reason may be as mentioned above by Hay *et al*,(24,25) that the incidence of bacterial vaginosis decreases with the increase in gestational age and may remit spontaneously. In this study, the lower incidence of bacterial vaginosis by Spiegel’s and Nugent’s criteria can be explained as most of women fell in the gestational age group from 21 to 30 weeks or they might had chronic infection in which clue cells were absent due to local immune response to IgA antibodies.

We found highly significant correlation of bacterial vaginosis with adverse incidences of poor pregnancy outcome. Out of 164 women who followed till the final outcome of delivery, 65 were having adverse outcome and the rest 99 were delivered without any complications [Graph 2]. Adverse outcomes such as preterm labor, PROM and fetal complications (prematurity, low birth weight) were found more in pregnant women with bacterial vaginosis(*N*=41), bacterial vaginosis with UTI (*N*=14) as compared to those without bacterial vaginosis (*N*=118) [Table 3]. The mechanism by which bacterial vaginosis causes the preterm birth of an infant with low birth weight is not known, but there is evidence that it causes infection of the upper genital tract, which in turn causes premature birth.(26) Pregnant women with bacterial vaginosis have elevated vaginal or cervical levels of endotoxin,(27) mucinase, sialidase,(28) and interleukin-1β,(27) suggesting that microorganisms that cause bacterial vaginosis stimulate the production of cytokines. A relative reduction in the number of vaginal lactobacilli is one characteristic of this syndrome,(15) further supporting the biologic plausibility of the hypothesis that bacterial vaginosis causes an increase in the preterm delivery of infants with low birth weight(29,30) in the present study out of 41 BV positive mothers, 25 having infants with low birth weight (LBW, <2.5 Kg) compared to 118 BV negative (18 infants) and only 4 infants LBW in BV with UTI [Table 3].

In the present study, the pregnancy outcome in pregnant women of bacterial vaginosis without UTI *vs* bacterial vaginosis with UTI were abortion(3 *vs* 1), PROM(11 *vs* 3), preterm labor (22 *vs* 9), puerperal pyrexia(2 *vs* 1) and low birth weight(25 *vs* 4 ) [Table 3]. It has been found that incidence of poor pregnancy outcome is higher in pregnant women having bacterial vaginosis without UTI than with UTI as the reason mentioned above that the diagnosis of UTI was made with mid-day sample of urine in OPD, because to avoid false results as patients were coming to our tertiary centre OPD so morning sample either get spoiled or some patients lost to follow-up.

Urinary tract infection and bacterial vaginosis are common coexisting conditions.(31–33) The incidence of UTI with bacterial vaginosis was also found higher by Harmauli *et al*.(34,35) In our study, the incidence of UTI were in 51/200 patients (found significant, *P*=0.000), incidence of UTI with bacterial vaginosis 14/41 and the incidence of UTI without bacterial vaginosis 37/159. The incidence of UTI with bacterial vaginosis is higher (34.1%) than without bacterial vaginosis (23.3%) (not significant, *P*=0.42) [Table 4].

## Conclusion

The incidence of poor pregnancy outcome was higher in bacterial vaginosis with UTI. Prevention of BV and UTI is cost effective to minimize the pregnancy outcome complication such as abortion, PROM, PPROM and preterm labor to decrease perinatal and maternal mortality and morbidity.
